# Nontyphoidal *Salmonella* Gastroenteritis in a Tertiary Children's Hospital in Southern China: Characteristics and Dietary Considerations

**DOI:** 10.1155/2018/3097468

**Published:** 2018-03-04

**Authors:** Lu Ren, Min Yang, Lanlan Geng, Peiyu Chen, Huan Chen, Sitang Gong, Ding-You Li

**Affiliations:** ^1^Department of Gastroenterology, Guangzhou Women and Children's Medical Center, Guangzhou Medical University, Guangzhou 510623, China; ^2^Division of Gastroenterology, Children's Mercy Kansas City, University of Missouri-Kansas City School of Medicine, Kansas City, MO 64108, USA

## Abstract

**Background:**

Nontyphoidal *Salmonella* infection is a common cause for acute bacterial gastroenteritis in children in China. There have been no reports of the prevalence of lactose intolerance or food allergies in children with nontyphoidal *Salmonella* infection. The aim of this study was to characterize nontyphoidal *Salmonella* gastroenteritis in a tertiary children's hospital and evaluate clinical presentation, lactose intolerance, and food allergies in children with prolonged nontyphoidal *Salmonella* gastroenteritis.

**Methods:**

A retrospective case-series analysis was carried out in a tertiary children's hospital in Guangzhou, China. We included all infants and children who were diagnosed with nontyphoidal *Salmonella* gastroenteritis between 1 January 2014 and 31 December 2016. Patients' clinical features, feeding patterns, laboratory tests, and treatment outcomes were reviewed.

**Results:**

A total of 142 infants and children were diagnosed with nontyphoidal *Salmonella* gastroenteritis. 52.1% of cases occurred in infants ≤ 12 months of age and the majority (89.4%) in children younger than 3 years old. The most common symptoms were diarrhea (100%), fever (62%), and vomiting (18.3%). *Salmonella* Typhimurium was the predominant serotype, accounting for 82.4%. 91.5% of patients were treated with antibiotics. Forty-one (28.9%) and 9 (6.3%) children improved with a lactose-free diet and hypoallergenic formula, respectively, when diarrhea persisted for more than a week.

**Conclusions:**

*Salmonella* Typhimurium was the predominant serotype. Most patients with nontyphoidal *Salmonella* gastroenteritis were younger than 3 years old. Lactose intolerance occurred frequently in children with nontyphoidal *Salmonella* gastroenteritis and dietary modification should be considered when diarrhea is persistent and prolonged.

## 1. Introduction

Nontyphoidal *Salmonella* infection is a common cause for acute bacterial gastroenteritis in children in China [1, 2]. The main symptoms of nontyphoidal *Salmonella* gastroenteritis in infants and children include acute onset of diarrhea, vomiting, fever, abdominal pain, and irritability. Nontyphoidal *Salmonella* gastroenteritis is mostly a self-limiting disease and resolves within one week in healthy children [3]. However, disease course may be prolonged in children with secondary lactase deficiency or food allergies [4–6]. There has been no study to assess the prevalence of lactose intolerance or food allergies in children with nontyphoidal *Salmonella* gastroenteritis. The aim of this study was to characterize nontyphoidal *Salmonella* gastroenteritis in a tertiary children's hospital in southern China and evaluate clinical presentation, lactose intolerance, and food allergies in children with prolonged nontyphoidal *Salmonella* gastroenteritis.

## 2. Methods

A retrospective case-series analysis was carried out in a tertiary children's hospital in Guangzhou, China. We reviewed all cases with stool culture performed and included all infants and children who were diagnosed with nontyphoidal *Salmonella* gastroenteritis between 1 January 2014 and 31 December 2016. Patients' clinical features, feeding patterns, laboratory tests, and treatment outcomes were reviewed. All children underwent routine evaluation including a complete blood count, electrolytes, C-reactive protein (CRP), blood culture for bacteria and fungus, stool culture for bacteria (*Escherichia coli*, *Salmonella, Shigella*, *Campylobacter*, and *Enterobacter aerogenes*), and stool viral antigen tests (rotavirus and adenovirus). All laboratory works were performed at our hospital laboratory, which is a standard tertiary hospital facility. The diagnosis of nontyphoidal *Salmonella* gastroenteritis was confirmed by stool culture. *Salmonella* isolates from stool samples were serotyped per standard protocol.

Treatments of children with nontyphoidal *Salmonella* gastroenteritis include general supportive care and antibiotic therapy if indicated [3]. Choices of antibiotics were guided by local antimicrobial resistance pattern and/or antimicrobial susceptibility testing. For patients with diarrhea that lasted for ≥7 days, stools were tested for carbohydrate malabsorption, and a lactose-free diet or formula was initiated if the stool reducing substance was positive and stool pH was <5.5 [7]. For children with confirmed or clinically suspected food allergies, hypoallergenic formula/diet would be initiated.

The institutional ethics committee of Guangzhou Women and Children's Medical Center approved this study protocol.

## 3. Results

There were 7379 stool cultures performed between 1 January 2014 and 31 December 2016. As shown in Table 1, a total of 142 infants and children were diagnosed with nontyphoidal *Salmonella* gastroenteritis, with male predominance. About half (52.1%) of them presented at <12 months of age. All infants presented with diarrhea 2–30 times/day with average 8 times/day. Most patients had mucus and blood in stools. Other common presenting symptoms included fever, vomiting, abdominal pain, bloating/distention, and irritability. Laboratory tests showed that most patients (89.4%) had elevated CRP. Leukocytosis, thrombocytosis, and anemia occurred in 42.3%, 40.8%, and 36.6% of children, respectively. Microscopic stool exam showed increased WBC and RBC in 62.7% and 33.1% of patients, respectively. Stool occult blood was positive in the majority (78.2%) of children.

Stool culture was positive for nontyphoidal *Salmonella* in all children. *Salmonella* Typhimurium was the predominant serotype, accounting for 82.4% of isolates. Other minor serotypes included Enteritidis, Saintpaul, Bovis mortificans, Thompson, Paratyphi B, and A.

Twenty-six children had prior infections including 20 (14.1%) with respiratory infection and 6 (4.2%) with hand, foot, and mouth disease.

All patients received supportive care including oral rehydration, probiotics (*Lactobacillus*/*Bifidobacterium* and/or *Saccharomyces boulardii*), and montmorillonite. 91.5% of patients were treated with antibiotics, with 73.2% administered intravenously. Forty-one (28.9%) children improved with lactose-free formula and/or diet when diarrhea persisted for more than a week and stool testing was positive for carbohydrate malabsorption. Five patients were tested positive for serum allergen-specific IgE (sIgE), and 4 patients were highly suspected of food allergy based on clinical symptoms although sIgE was negative. All 9 patients (6.3%) improved with hypoallergenic formula (extensively hydrolyzed or amino-acid formula).

All children had clinical resolution with majority (90.8%) within 2 weeks. No significant adverse reactions were observed.

## 4. Discussion

Nontyphoidal *Salmonella* is a major enteric pathogen for acute bacterial gastroenteritis in children in China [1, 8, 9]. In our study, about half of cases occurred in infants ≤ 12 months of age and the majority (89.4%) in children younger than 3 years old, consistent with other reports [1, 2]. The main symptoms in our cohort were diarrhea, fever, and vomiting. Most infants and children had mucus and/or blood in stools. Laboratory study revealed that the majority (89.4%) of patients had elevated CRP while leukocytosis, thrombocytosis, and anemia occurred in less than 50% of cases.

In our study, nontyphoidal *Salmonella* Typhimurium was the predominant (82.4%) serotype, followed distantly by Enteritidis (8.5%), Saintpaul (4.2%), bovis morbificans (1.4%), Thompson (1.4%), Paratyphi B (1.4%), and Paratyphi A (0.7%). Our results were different from those reported by others in China. Ran et al. reported that serotype Enteritidis (31%) and serotype Typhimurium (26%) were the most common, and Li et al. found that Enteritidis (38.9%) and Typhimurium (29.7%) were the most common serotypes [1, 9]. The inconsistency may be due to the changing epidemiological trend, the regional variation, and the specific patient population. As a tertiary children's center, the patients in this study were referred from surrounding hospitals due to persistent symptoms in spite of supportive care and other treatments. It would be interesting to investigate the nontyphoidal *Salmonella* serotype in those surrounding hospitals.

Antibiotic therapy is generally not recommended for uncomplicated gastroenteritis in healthy children with nontyphoidal *Salmonella* infection [3]. In our study, the majority (91.5%) of infants and children received oral or intravenous antibiotics. This is likely due to our specific patient population referred from surrounding hospitals where initial supportive care failed. With appropriate antibiotic therapy and other supportive care, the majority (90.8%) of our patients had clinic resolution within 2 weeks.

Early studies suggest that secondary lactose intolerance and food allergies occur in children with acute infectious diarrhea, and lactose-free or hypoallergenic formulas improve symptoms [4, 5, 10]. However, there has been no any reports of the prevalence of lactose intolerance or food allergies in children with nontyphoidal *Salmonella* infection. Our study showed that 28.9% of children had significant symptom improvement with a lactose-free formula or diet, indicating that secondary lactase deficiency occurs frequently in children with nontyphoidal *Salmonella* gastroenteritis. Only 6.3% had confirmed or suspected food allergies. Dietary modification should be considered when diarrhea is persistent and prolonged.

The major strength of this study is that this is the first report of relatively common secondary lactase deficiency in children with nontyphoidal *Salmonella* gastroenteritis. However, this study has weaknesses and limitations. This is a retrospective case review, and a standard lactose breath test was not used to diagnose lactose intolerance. A larger epidemiology study would be necessary to assess the true prevalence of lactose intolerance and food allergies in children with nontyphoidal *Salmonella* infection.

## 5. Conclusions

This study demonstrates that *Salmonella* Typhimurium was the predominant serotype. Most patients with nontyphoidal *Salmonella* gastroenteritis are younger than 3 years old, and the main symptoms are diarrhea, fever, and vomiting. Lactose intolerance occurs frequently in children with nontyphoidal *Salmonella* gastroenteritis. Dietary modification should be considered when diarrhea is persistent and prolonged.

## Figures and Tables

**Table 1:** Basic characteristics, clinical features, and laboratory tests and treatments in 142 infants and children with nontyphoidal *Salmonella* gastroenteritis.

Age	
<12 months	74 (52.1%)
1–3 years	53 (37.3%)
≥3 years	15 (10.6%)
Sex, M/F	64.1%/35.9%
Disease duration at presentation	
<2 weeks	100 (70.4%)
2–4 weeks	31 (21.8%)
≥4 weeks	11 (7.8%)
Prior illness	
Respiratory infection	20 (14.1%)
Hand, foot, and mouth disease	6 (4.2%)
Symptoms	
Diarrhea	142 (100%)
Mucus in stool	85 (59.9%)
Blood in stool	76 (53.5%)
Fever (T ≥ 38°C)	88 (62.0%)
Nausea and vomiting	26 (18.3%)
Bloating/distention	10 (7.0%)
Abdominal pain	10 (7.0%)
Irritability	5 (3.5%)
Laboratory studies	
Leukocytosis (≥12 × 10^9^/L)	60 (42.3%)
Thrombocytosis (≥300 × 10^9^/L)	58 (40.8%)
Anemia (HB< 110 g/L)	52 (36.6%)
CRP (≥1 mg/L)	127 (89.4%)
Stool WBC ≥ (++)	89 (62.7%)
Stool RBC ≥ (++)	47 (33.1%)
Stool occult blood (+)	111 (78.2%
Stool culture: positive Salmonella	142 (100%)
Salmonella serotype	
Typhimurium	117 (82.4%)
Enteritidis	12 (8.5%)
Saintpaul	6 (4.2%)
bovis morbificans	2 (1.4%)
Thompson	2 (1.4%)
Paratyphi B	2 (1.4%)
Paratyphi A	1 (0.7%)
Treatments	
Antibiotics	130 (91.5%)
Oral antibiotics	26 (18.3%)
IV antibiotics	104 (73.2%)
Lactose-free formula/diet	41 (28.9%)
Hypoallergenic formula	9 (6.3%)
Clinical resolution after treatment	
<2 weeks	129 (90.8%)
2–4 weeks	9 (6.3%)
>4 weeks	4 (2.8%)

## Data Availability

The data supporting the current findings are not publicly available since the database is currently not anonymous and contains all the patients' names. However, it will be available upon request.
